# A rare case of epiploic appendagitis of the vermiform appendix

**DOI:** 10.1093/jscr/rjag456

**Published:** 2026-09-21

**Authors:** Van Trung Hoang, Hien Nguyen, Hoang Anh Van, The Huan Hoang, Vichit Chansomphou, Duc Thanh Hoang

**Affiliations:** Department of Radiology, Thien Hanh Hospital, Dak Lak, Vietnam; Department of Radiology, Thien Hanh Hospital, Dak Lak, Vietnam; Department of Radiology, Thien Hanh Hospital, Dak Lak, Vietnam; Department of Radiology, Thien Hanh Hospital, Dak Lak, Vietnam; Department of Radiology, Savannakhet Medical-Diagnostic Center, Kaysone Phomvihane, Lao People’s Democratic Republic; Division of Endocrinology, Department of Medicine, Walter Reed National Military Medical Center, Bethesda, MD, United States; Division of Endocrinology, Uniformed Services University of the Health Sciences, Bethesda, MD, United States

**Keywords:** acute abdomen, appendicitis mimic, gastroenterology, epiploic appendagitis, vermiform appendix

## Abstract

Epiploic appendagitis is a rare and often overlooked cause of acute abdominal pain that can mimic more common surgical conditions such as acute appendicitis or diverticulitis. Most reported cases involve the sigmoid or descending colon, whereas epiploic appendagitis occurring near the vermiform appendix is extremely rare. We report the case of a 57-year-old woman who presented with acute right lower quadrant pain initially suspected to be acute appendicitis. Computed tomography revealed inflammation of the epiploic appendage adjacent to the base of the appendix, with mild reactive appendicitis. The patient underwent surgery successfully, which confirmed torsion and necrosis of the appendiceal epiploic appendage. Early and accurate diagnosis of this condition is crucial to guide appropriate management.

## Introduction

The term epiploic appendagitis was introduced by Lynn *et al.* and refers to an uncommon diagnosis characterized by the sudden onset of localized pain in the left or right lower abdominal quadrant [1]. Epiploic appendagitis is a rare inflammatory or ischemic process affecting the epiploic appendages of the colon, which may represent either a primary condition or occur secondary to adjacent pathology. Torsion of an epiploic appendage is a complication that can arise from either inflammatory or non-inflammatory processes [2, 3]. Normally, there are ~50 to 100 epiploic appendages distributed along the colon, extending from the cecum to the sigmoid-rectal junction, and they are covered by peritoneum. These structures are finger-like in shape, measuring 2 to 5 cm in length and 1 to 2 cm in thickness, with the largest appendages typically found along the descending and sigmoid colon [4, 5]. Several physiological functions have been suggested for the epiploic appendages, including serving as a supportive cushion for the colon, participating in immune responses similar to the small omentum, and being involved in the absorptive processes of the colon. Each epiploic appendage receives its blood supply from 1 or 2 small arteries and is drained by a single vein passing through its narrow stalk. This limited blood supply, combined with the pedunculated shape and mobility of the appendages, makes them highly prone to torsion, potentially leading to infarction or hemorrhage. There is a higher prevalence among women and obese individuals, possibly due to the presence of larger epiploic appendages in these groups [2, 3, 5, 6].

Epiploic appendagitis most commonly involves the sigmoid and descending colon, whereas involvement near the vermiform appendix is extremely rare. Although the true incidence remains unclear, appendiceal epiploic appendagitis has been reported in ~0.3% to 1% of patients initially suspected of acute appendicitis and accounts for only a small proportion of all epiploic appendagitis cases. Due to its rarity and nonspecific presentation, it is frequently misdiagnosed as acute appendicitis [2, 4, 7, 8].

To contribute to the understanding of and existing literature on this rare condition, we report a case of a 57-year-old woman who presented with acute right lower quadrant abdominal pain, initially suspected to be acute appendicitis. Ultrasound suggested possible appendicitis. Contrast-enhanced computed tomography (CT) revealed epiploic appendagitis adjacent to the base of the appendix, with mild inflammatory changes in the appendix itself. Surgical findings confirmed torsion and necrosis of the appendiceal epiploic appendage.

## Case report

A 57-year-old woman presented to our emergency department with a 2-day history of dull pain localized to the right iliac fossa, which worsened with coughing and movement. She had no fever, nausea, vomiting, changes in bowel habits, or urinary symptoms. Her medical history was unremarkable, with no prior abdominal surgery or trauma. She was not taking any medication and had no known drug allergy.

Clinical examination revealed a temperature of 37.5°C, a heart rate of 82 beats/minute, and a blood pressure of 125/70 mmHg. Abdominal examination demonstrated localized tenderness in the right lower abdominal quadrant without rebound, guarding, or palpable mass, and bowel sounds were normal. Laboratory tests showed a white blood cell count of 5200/mm^3^ (reference 4500–11 000) and a C-reactive protein (CRP) level of 3.2 mg/L (reference <0.3). Liver and renal function tests, as well as urinalysis, were unremarkable.

**Figure 1:**
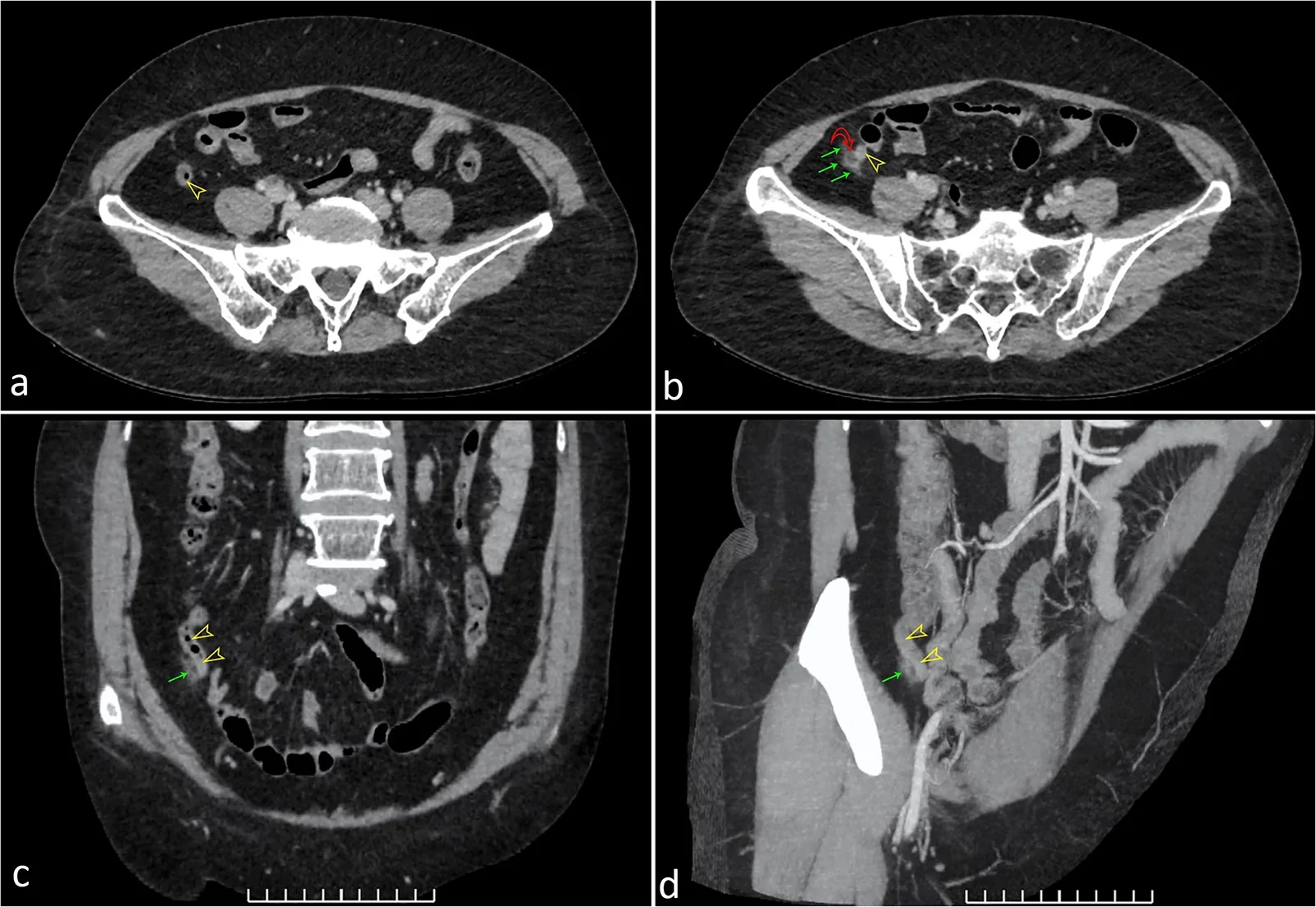
Contrast-enhanced CT images in (a, b) axial, (c) coronal, and (d) oblique coronal planes with MIP reconstruction show a mildly enlarged appendix (arrowheads), containing fluid and gas. Adjacent to the distal portion of the appendiceal body, there is a round, fat-density structure (curved arrow) with mild surrounding fat stranding (small arrows).

Abdominal ultrasound demonstrated an appendix measuring ~5 to 6.5 mm in diameter with mild surrounding fat stranding, suggestive of early acute appendicitis. Because the clinical and ultrasound findings were inconclusive for a definitive diagnosis, a contrast-enhanced abdominal CT scan was performed, revealing a mildly enlarged appendix measuring ~6 to 7 mm in diameter, containing fluid and gas within its lumen, with mild wall thickening and slight enhancement. In addition, a well-defined, oval, fat-density lesion measuring ~4 × 5 mm was identified at the distal portion of the vermiform appendix (Fig. 1). This fatty structure exhibited a hyperattenuating rim (hyperattenuating ring sign) with mild surrounding fat stranding. No peritoneal free fluid or free air was observed. These findings were consistent with epiploic appendagitis of the appendix and reactive appendicitis.

**Figure 2:**
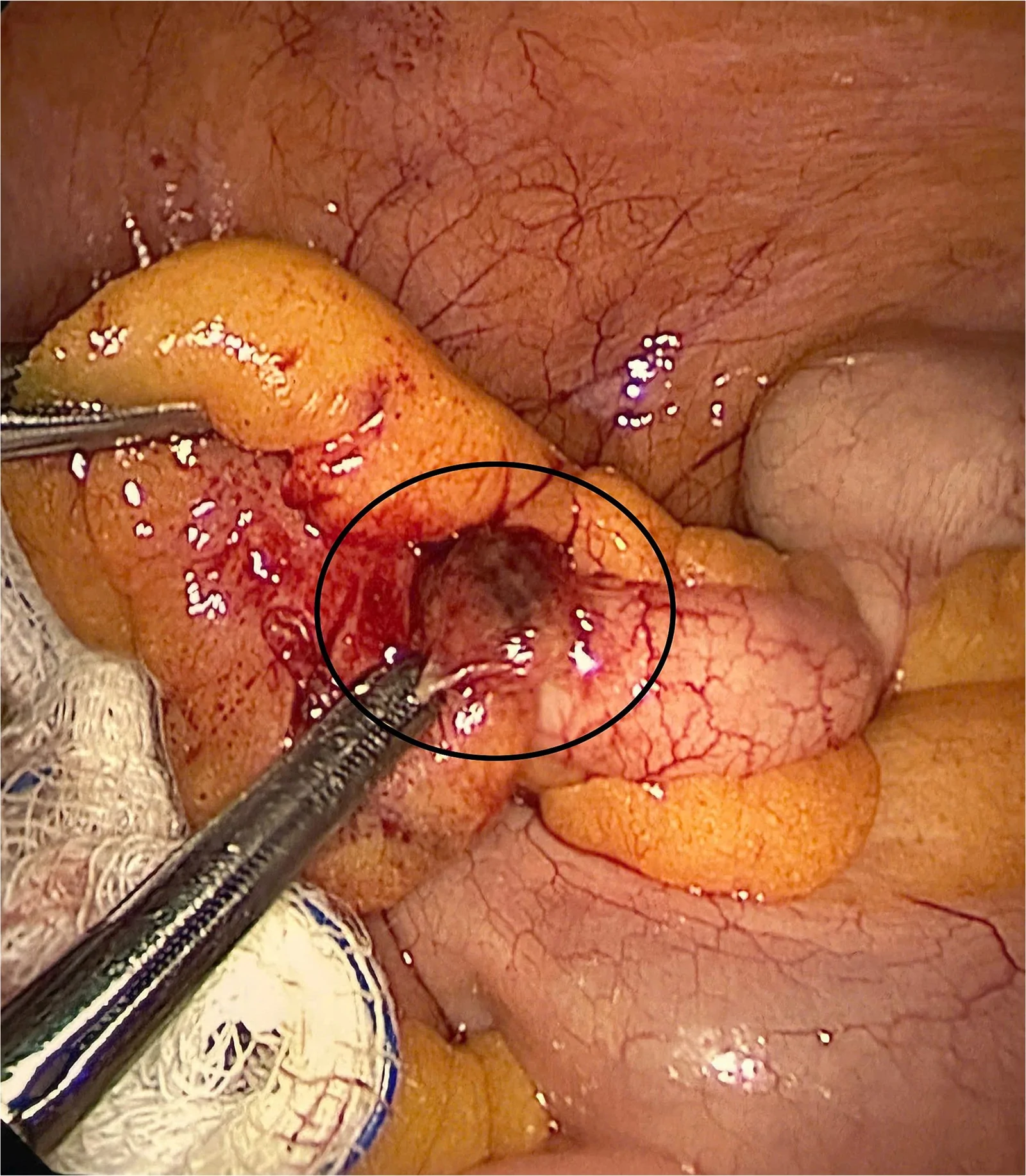
Intraoperative view showing a torsioned and necrotic epiploic appendage of the appendix (circled), along with a hyperemic, inflamed appendix.

The patient subsequently underwent laparoscopic surgery under general anesthesia in the supine position. A 1-cm midline incision was made above the umbilicus, the linea alba was opened, and a 10-mm trocar was inserted to establish pneumoperitoneum. Two additional trocars were placed: a 5-mm trocar in the lower epigastrium and a 10-mm trocar in the left lower quadrant. Intraoperative exploration revealed a swollen appendix with pus, torsion, and necrosis of the mesoappendiceal fat (Fig. 2 and Video S1). The mesoappendix was divided using bipolar electrocautery. The base of the appendix was ligated with a Roeder knot, and the appendix was transected. The peritoneal cavity was irrigated, and no active bleeding was observed. The appendix was retrieved through the 10-mm trocar site using a specimen bag. Pneumoperitoneum was released, trocars were removed, and port sites were closed.

She was discharged home on postoperative day 3 and remained asymptomatic at the 2-week follow-up.

## Discussion

Appendiceal epiploic appendagitis typically presents with acute, continuous, non-radiating pain localized to the right lower abdominal quadrant. Because the epiploic appendages are covered by visceral peritoneum, inflammation leads to localized peritoneal irritation surrounding the affected appendage, often resulting in more well-localized pain compared to other causes of abdominal pain. The pain characteristically does not exhibit the progressive intensification often seen in acute appendicitis. On physical examination, localized tenderness is observed, without significant peritoneal signs or marked guarding [1–3].

The most common mechanism leading to appendiceal epiploic appendagitis is acute torsion of abnormally long and large epiploic appendages, causing ischemia and necrosis. Primary thrombosis of the central draining vein of the omentum is also one of the potential causes of this condition. Additional risk factors that have been reported include obesity, sudden or vigorous physical movements, and middle age [2–5].

Appendiceal epiploic appendagitis often poses a diagnostic challenge due to its nonspecific clinical presentation and symptoms that closely mimic those of acute appendicitis. Therefore, the use of imaging modalities plays a crucial and often decisive role in diagnosing this rare condition [4, 5].

On ultrasound, epiploic appendagitis generally appears as a round or oval, tender, non-compressible mass measuring ~1.5 to 3.5 cm. It typically demonstrates a central area of hyperechoic, heterogeneous fat infiltration surrounded by a hypoechoic rim due to peritoneal inflammation around the appendage. There may also be inflammatory fat stranding surrounding the affected appendage, and mild thickening of adjacent bowel loops due to a reactive process. Occasionally, the central region of the lesion may appear hypoechoic due to intravascular thrombosis. Color Doppler examination usually shows no significant vascularity within the lesion.

In cases where clinical and ultrasound findings are equivocal or inconclusive, contrast-enhanced abdominal CT is essential for confirming the diagnosis and differentiating it from other pathologies. Thus, CT is considered the preferred diagnostic modality for appendiceal epiploic appendagitis [5, 6, 8].

Characteristic CT features of appendiceal epiploic appendagitis include a round or oval fat-density lesion, usually small (4–15 mm), with a thin hyperattenuating rim that enhances after intravenous contrast administration, representing the inflamed visceral peritoneal layer—a finding known as the hyperattenuating ring sign [3, 7]. There may also be surrounding inflammatory fat stranding, and occasionally a central hyperattenuating dot, representing a thrombosed vein, known as the central dot sign [2, 5, 8]. In addition, the appendix may appear normal or may demonstrate mild wall thickening or enhancement due to a reactive process, without significant mural thickening, helping distinguish it from acute appendicitis or diverticulitis [9, 10].

Beyond characteristic CT findings, differentiating between appendiceal epiploic appendagitis and acute appendicitis based on clinical symptoms and laboratory results is also crucial in patients presenting with localized right lower quadrant abdominal pain. Patients with acute appendicitis frequently exhibit fever, leukocytosis, elevated C-reactive protein level of 3.2 mg/L (reference <0.3), nausea, vomiting, and initially dull right lower quadrant abdominal pain that progressively intensifies. On examination, there is often significant abdominal guarding and rebound tenderness. By contrast, appendiceal epiploic appendagitis generally manifests with acute, continuous, non-radiating right lower quadrant pain without accompanying systemic symptoms such as fever, anorexia, nausea, vomiting, diarrhea, or constipation. Physical examination of patients with epiploic appendagitis reveals localized tenderness without significant peritoneal signs. Laboratory tests are often normal or may show only mild leukocytosis [6, 7, 10].

In our case, the patient had a normal white blood cell count (5200/mm^3^) and only mildly elevated C-reactive protein levels, which further supports the diagnosis of epiploic appendagitis rather than acute appendicitis. This relatively unremarkable laboratory profile is a helpful distinguishing feature, as acute appendicitis is more commonly associated with leukocytosis and significant inflammatory marker elevation.

Appendiceal epiploic appendagitis is a self-limiting condition, with most patients recovering spontaneously within 1 to 14 days with analgesic, anti-inflammatory, and occasionally antibiotic therapy. Surgery is considered for patients unresponsive to conservative treatment or those with new symptoms, worsening conditions, or complications. The decision between conservative and surgical management can sometimes be challenging, as epiploic appendagitis may be complicated by ischemia and necrosis due to torsion. Surgical treatment typically involves resection of both the affected epiploic appendage and the appendix [8, 9, 11].

In our case, if the condition had been managed non-surgically, the torsioned and necrotic epiploic appendage could have autoamputated and transformed into a loose intraperitoneal body.

From a historical perspective, Virchow first proposed in 1853 that detached epiploic appendages could evolve into loose intraperitoneal bodies, which may subsequently calcify [2, 3, 5]. In cases of untreated torsion and necrosis, the affected appendage may undergo autoamputation and persist as a free peritoneal body. This mechanism reflects the natural evolution of epiploic appendagitis when managed conservatively and highlights the potential outcomes of non-surgical management [4, 7, 8, 11].

Recurrence of epiploic appendagitis is rare. However, a tendency for recurrence in patients managed conservatively has been reported in ~40% of cases [7, 8, 10]. This raises concerns that conservative treatment may predispose to recurrence, and surgical intervention should be considered. For epiploic appendagitis occurring in other parts of the colon, conservative management is generally the first-line treatment. However, in cases of appendiceal epiploic appendagitis, surgery should be considered as the initial approach due to potential concerns about the associated complications of acute appendicitis [9, 11, 12]. In our case, it was uncertain whether conservative treatment would have been effective, but surgical management with excision of the inflamed epiploic appendage and appendectomy resulted in complete recovery for the patient. Several authors advocate for laparoscopic removal of the affected epiploic appendages together with a prophylactic appendectomy in such cases, regardless of whether the appendix appears normal [13, 14].

## Conclusion

Appendiceal epiploic appendagitis represents an uncommon yet significant differential diagnosis in patients presenting with right lower quadrant abdominal pain. Recognizing its clinical presentation and imaging characteristics allows for accurate diagnosis and appropriate conservative or surgical treatment strategies.

## Supplementary Material

Video_S1_rjag456 Intraoperative video demonstrating appendiceal epiploic appendagitis.
